# The value of ventricular measurements in the prediction of shunt dependency after aneurysmal subarachnoid hemorrhage

**DOI:** 10.1007/s00701-023-05595-6

**Published:** 2023-05-02

**Authors:** Maryam Said, Meltem Gümüs, Jan Rodemerk, Mehdi Chihi, Laurèl Rauschenbach, Thiemo F. Dinger, Marvin Darkwah Oppong, Philipp Dammann, Karsten H. Wrede, Ulrich Sure, Ramazan Jabbarli

**Affiliations:** 1 Department of Neurosurgery and Spine Surgery, University Hospital of Essen, Essen, Germany; 2 Department of Neurosurgery and Spine Surgery, Evangelisches Krankenhaus Oldenburg, Oldenburg, Germany; 3 Center for Translational Neuro- & Behavioral Sciences (C-TNBS), University Duisburg-Essen, Essen, Germany

**Keywords:** Ventricular measurements, Chronic hydrocephalus, Shunt dependency

## Abstract

**Objective:**

Chronic hydrocephalus requiring shunt placement is a common complication of aneurysmal subarachnoid hemorrhage (SAH). Different risk factors and prediction scores for post-SAH shunt dependency have been evaluated so far. We analyzed the value of ventricle measurements for prediction of the need for shunt placement in SAH patients.

**Methods:**

Eligible SAH cases treated between 01/2003 and 06/2016 were included. Initial computed tomography scans were reviewed to measure ventricle indices (bifrontal, bicaudate, Evans’, ventricular, Huckman’s, and third ventricle ratio). Previously introduced CHESS and SDASH scores for shunt dependency were calculated. Receiver operating characteristic analyses were performed for diagnostic accuracy of the ventricle indices and to identify the clinically relevant cut-offs.

**Results:**

Shunt placement followed in 221 (36.5%) of 606 patients. In univariate analyses, all ventricular indices were associated with shunting (all: *p*<0.0001). The area under the curve (AUC) ranged between 0.622 and 0.662. In multivariate analyses, only Huckman’s index was associated with shunt dependency (cut-off at ≥6.0cm, *p*<0.0001) independent of the CHESS score as baseline prediction model. A combined score (0–10 points) containing the CHESS score components (0–8 points) and Huckman’s index (+2 points) showed better diagnostic accuracy (AUC=0.751) than the CHESS (AUC=0.713) and SDASH (AUC=0.693) scores and the highest overall model quality (0.71 vs. 0.65 and 0.67), respectively.

**Conclusions:**

Ventricle measurements are feasible for early prediction of shunt placement after SAH. The combined prediction model containing the CHESS score and Huckman’s index showed remarkable diagnostic accuracy regarding identification of SAH individuals requiring shunt placement. External validation of the presented combined CHESS-Huckman score is mandatory.

**Supplementary Information:**

The online version contains supplementary material available at 10.1007/s00701-023-05595-6.

## Introduction

Subarachnoid hemorrhage (SAH) after intracranial aneurysm rupture is known to have several early and delayed complications strongly impacting the further course and outcome of disease [26]. Post-hemorrhagic hydrocephalus is one of these common SAH complications, with early onset as an acute hydrocephalus affecting up to 97% of individuals [16]. However, not all these patients require a permanent cerebrospinal fluid (CSF) diversion, and the rate of the reported shunt dependencies in SAH series is about a third of the cases [16, 27]. To reduce the weaning time of external ventricular drains (EVD), timely selection of the patients in need of a shunt is of clinical relevance [28]. Identification could help prevent CSF infections [15] and shorten the duration of hospital stay [7].

Several clinical studies have already focused on identifying early predictors of shunt dependency after SAH [14, 16, 27]. In order to increase the diagnostic accuracy of prediction of shunt dependency, several authors also attempted the construction of risk scores for the identification of SAH individuals requiring shunt placement, such as the chronic hydrocephalus ensuing from SAH Score (CHESS) [27] and the shunt dependency in SAH (SDASH) [14] scores. These scores are based on well-known risk factors like poor initial clinical condition, acute hydrocephalus, amount and pattern of intracranial bleeding, aneurysm location, and development of cerebral infarction. However, there is still a non-negligible risk of diagnostic inaccuracy of these scores limiting their implementation in the clinical routine.

Therefore, identifying novel risk factors for chronic hydrocephalus after SAH will aid in further improvement of the predictive power of the risk scores for post-SAH shunt dependency. In this context, the morphology of the ventricles might present a potential clinical value. Ventricular enlargement and other changes in ventricle morphology have been described in several other neurological disorders. In particular, the Evans’ index introduced in 1942 was used to diagnose normal pressure hydrocephalus and until now remains included in the criteria of patients’ selection [37, 48]. Also, the clinical value of the ventricular indices in Morbus Alzheimer [31] and multiple sclerosis [2] was reported. As to acute neurological conditions, ventricular enlargement described for traumatic brain injury (neonatal), intraventricular hemorrhage (IVH) and SAH, has been linked with increased morbidity [5]. Finally, there are only few studies on the association between ventricular measurements and shunt dependency after SAH [41, 51]. However, these studies are based on small SAH cohorts and do not compare the diagnostic value of different ventricular indices.

In light of the above-mentioned evidence on the clinical value of ventricle indices, we aimed to analyze different parameters of ventricular morphology concerning early prediction of chronic hydrocephalus necessitating shunt placement in SAH patients. A special emphasis was put on the evaluation of the additive predictive value of ventricular indices in the context of currently available SAH shunt prediction scores.

## Methods

### Patient population

In this retrospective study, we included all eligible consecutive SAH patients treated at our institution between January 2003 and June 2016. Patients were adults (>18 years) and had an available pre-treatment computer tomography (CT) scan <48h after ictus, enabling the measurement of the different ventricular parameters and survived long enough to evaluate the need for shunt placement. Patients were excluded from the study if they (a) did not receive aneurysm treatment, were (b) transferred elsewhere or (c) deceased before the end of weaning of CSF diversion, and (d) were admitted >48h after ictus or did not have an available CT scan <48h after ictus. The local ethics committee (Ethik-Kommission, Medizinische Fakultät der Universität Duisburg-Essen, Registration number: 15-6331-BO) approved our study. It was conducted within the clinical trial registered at the German trial registry (DRKS, Unique identifier: RKS00008749).

### SAH management

To confirm the rupture of an intracranial aneurysm, all patients underwent digital subtraction angiography (DSA) and were admitted to our neurosurgical intensive care unit. Aneurysms were secured by microsurgical clipping or endovascular coiling, mostly within 24 h after admission. Post-interventional intensive care treatment of SAH patients in our center was already described elsewhere [11, 43] [10, 42]. In short, conservative management consisted of maintenance of normovolemia, mean arterial pressure >70 mmHg, and oral nimodipine for the first 21 days after ictus. Vasospasm surveillance included daily transcranial Doppler ultrasonography with repeated DSA for vasospasm verification and endovascular treatment in SAH patients suspected of symptomatic vasospasm. In case of acute hydrocephalus, an EVD was inserted. If no contraindications such as increased intracranial pressure (ICP) or meningitis were present, patients were weaned from the external CSF drainage after 1 week by closing the EVD for 48 h. Prior to and after disengagement of the EVD, routine CT scans were performed. The weaning was considered successful when patients (a) did not have pathologically increased ICPs (>20 mmHg) in this period; (b) developed no neurological deterioration and/or increased headache reversible by opening of the drain; and (c) showed no increase of ventricle width in the post-challenge CT scan. Additional CT scans were performed with any neurological deterioration, after every surgical intervention, and before and after placement of a ventriculoperitoneal shunt. The indication for shunt placement was based on the presence of above-mentioned clinical and radiographic signs of chronic hydrocephalus and the failure of previous EVD weaning attempts. In SAH patients suspected of chronic hydrocephalus without EVD, a lumbar puncture with measurement of opening pressure was performed.

### Data management

**Fig. 1:**
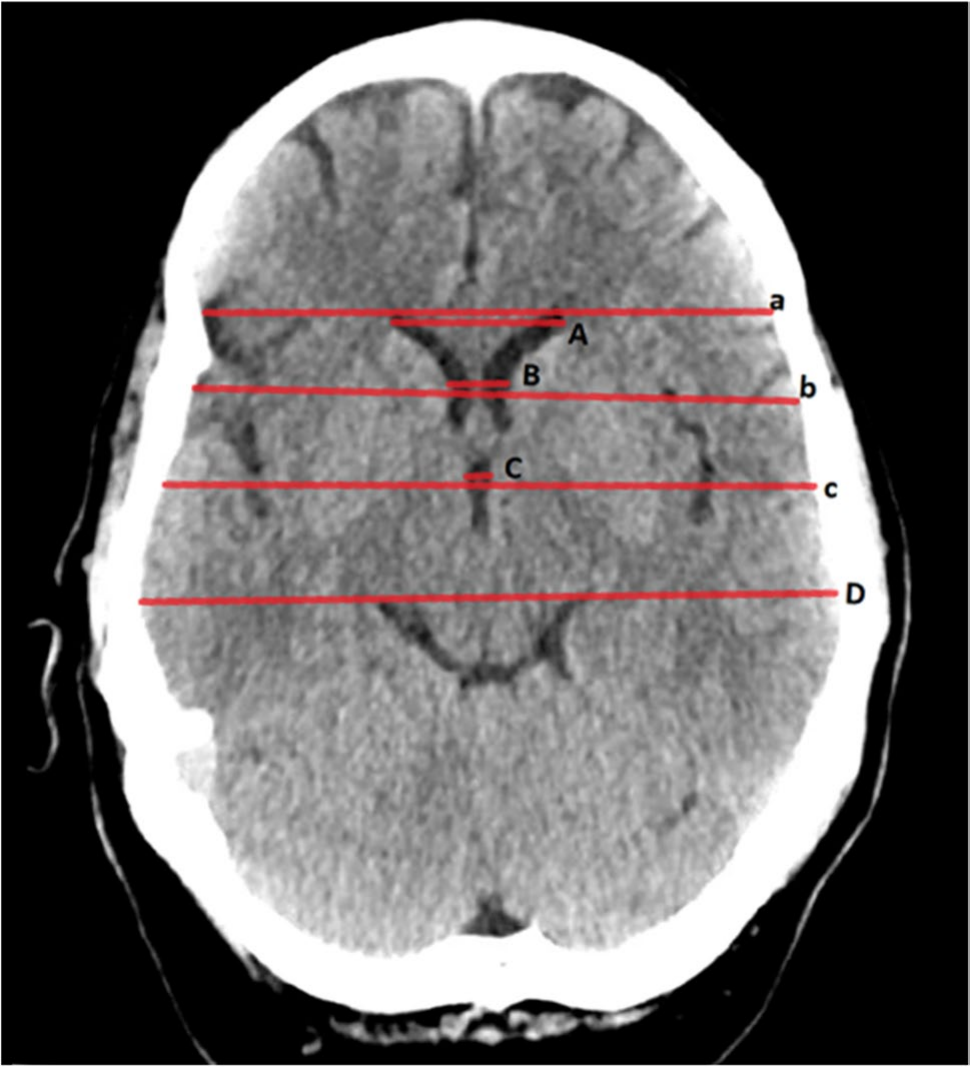
Illustration of the ventricular measurements. Bifrontal ratio: maximum width between the two frontal horns (A)/internal width of the vault at same level (a). Bicaudate ratio: minimum width of the ventricles between caudate nuclei (B)/internal width of the vault at same level (b). Ventricular ratio: minimum width of the ventricles (B)/maximum width between frontal horns (A). Third ventricle ratio: greatest width of the third ventricle (C)/internal width of the vault at same level (c). Evans’ index: maximum width between frontal horns (A)/maximum internal width of the vault (D). Huckman’s index: maximum width between the two frontal horns (A) + minimum width of the ventricles between caudate nuclei (B)

For this study, the first available pre-treatment CT scan after admission was reviewed by the first author (M.S.), blinded at this time for any clinical information. The components of the bifrontal, bicaudate, ventricular and third ventricle ratios, and Evans’ and Huckman’s indices were measured, as described in previous literature [3, 17, 20, 22, 32, 39]. A graphic depiction of the different measurements is provided in Fig. 1. Other radiographic parameters assessed with the DSA and CT scans were the location of the ruptured aneurysm, presence of IVH (defined as any evidence of acute intraventricular bleeding visualized in the initial CT scan), the Barrow Neurological Institute (BNI) grading scale [52] at admission, and the development of early cerebral infarcts within 72 h post-SAH according to previous data assessment [29]. To calculate the CHESS and SDASH scores (see supplementary Table S1 in Online Supplements) and record the rates of post-SAH hydrocephalus, initial clinical condition according to the Hunt and Hess scale [24], as well as acute and chronic hydrocephalus requiring shunt placement, was also collected for further analysis. The presence of acute hydrocephalus was judged upon the specific radiographic (ventricle enlargement on the basis of the third ventricle width and periventricular low density on CT scan) and clinical signs (such as mental deterioration, memory impairment, gait disturbance, and urinary incontinence) occurring within 3 days after ictus [1, 14, 27]. To address the possible discrepancy between the true shunt dependency and documented shunt placement rates (due to prophylactic shunt implantation), the whole post-SAH treatment data from the electronic medical records were reviewed with regard to the need for shunt revision or removal surgery due to shunt over-drainage.

### Study endpoint and statistical analyses

The purpose of our study was to analyze the additive predictive value of the different ventricular measurements in the context of previously reported shunt dependency risk scores for SAH patients. First, the associations between the ventricular morphology parameters and shunt placement were evaluated in univariate analysis using the Student’s *t* test for normally distributed continuous data and the Mann–Whitney *U* test for non-normally distributed continuous data. The significant associations were tested in the receiver operating characteristic (ROC) curves to elucidate the diagnostic accuracy of and identify the clinically relevant cut-off points for all selected ventricular indices. Then, a multivariate backward stepwise regression analysis was performed. As the baseline prediction model, it included the currently available risk score with the highest value of the area under the curve (AUC) in the ROC analysis (the CHESS score). Moreover, the backward stepwise regression analysis included all ventricular parameters as dichotomous variables (according to the ROC-based cut-off values). Finally, the significant results from this multivariate analysis were used for the construction of a novel combined risk score for the prediction of shunt placement after SAH. The weights of the score components were calculated using the values of the adjusted odds ratios (aOR). The significant aORs were divided by the smallest coefficient and rounded to the nearest whole number. After calculating the novel risk score values for all individuals in the cohort, its diagnostic accuracy for the prediction of shunt placement was assessed and compared with the previously introduced risk scores using the ROC analyses: the AUC of the ROC curve, precision–recall curve, and overall model quality.

Data analysis was performed using SPSS statistical software (version 25, SPSS Inc., IBM). Correlations with a *p* value of ≤0.05 were considered statistically significant.

## Results

**Fig. 2:**
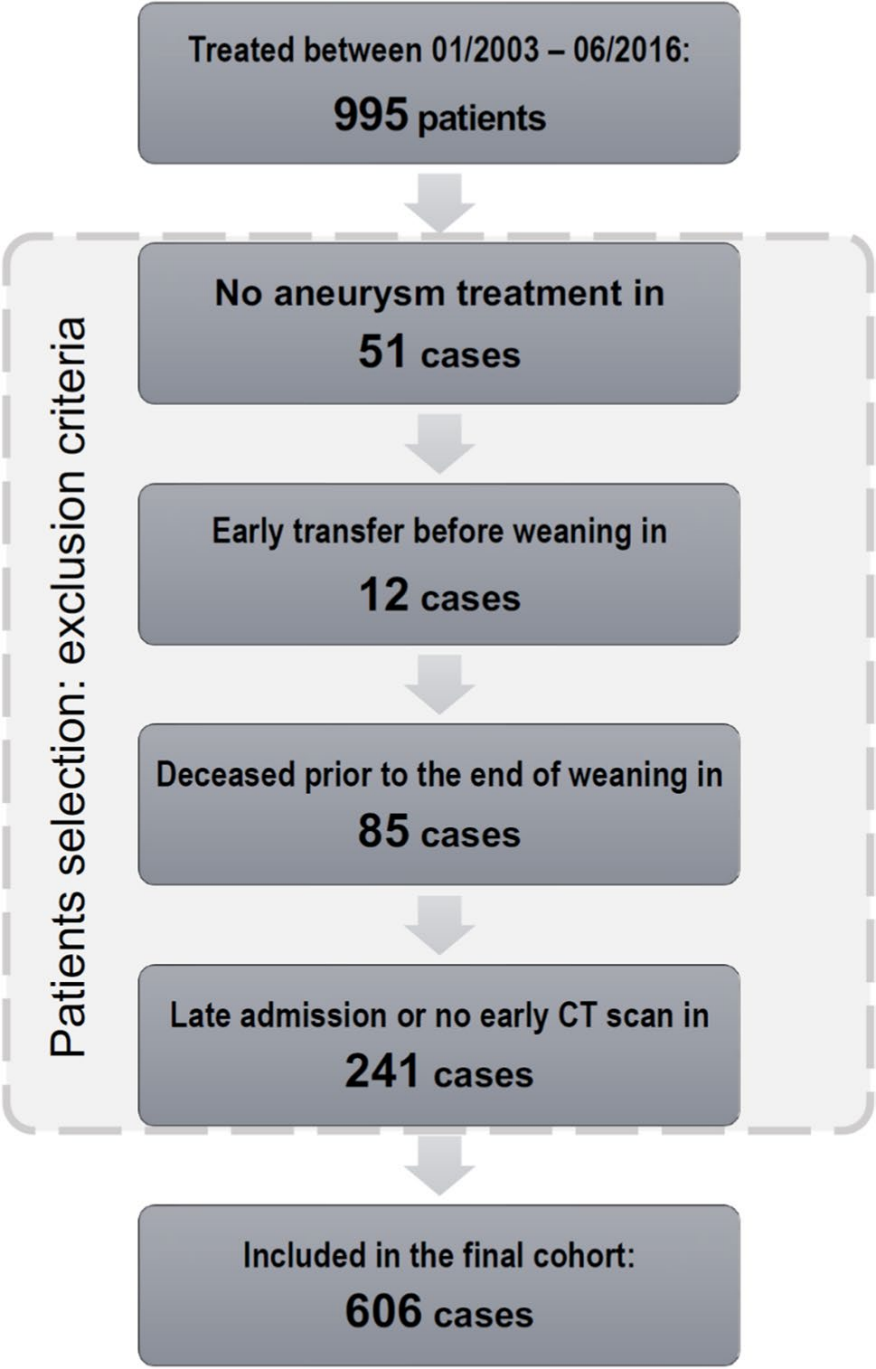
Flowchart of the included SAH patients in the final cohort. Exclusion criteria and number of patients per criterion are depicted in the chart

Of the 995 patients treated at our institution in the mentioned study period, we could include a total of 606 cases in the final cohort after applying the exclusion criteria (see Fig. 2 for the flowchart). An overview of the study-related patients’ characteristics is provided in Table 1.

**Table 1:** Ventricular measurements and patients’ characteristics

Parameter	Number of cases (%) or mean (±SD)	
	Shunt placed (*n*=221)	No shunt (*n*=385)
Ventricular measurements		
A (cm)	3.77 (±0.62)	3.46 (±0.56)
B (cm)	2.05 (±0.69)	1.70 (±0.54)
C (cm)	0.84 (±0.37)	0.69 (±0.66)
Bifrontal ratio: A/a	0.35 (±0.06)	0.33 (±0.05)
Bicaudate ratio: B/b	0.18 (±0.06)	0.15 (±0.10)
Ventricular ratio: B/A	0.54 (±0.12)	0.49 (±0.11)
Third ventricle ratio: C/c	0.07 (±0.03)	0.06 (±0.06)
Evans’ index: A/D	0.29 (±0.05)	0.27 (±0.04)
Huckman’s index: A+B (cm)	5.77 (±1.32)	5.14 (±1.08)
Shunt dependency risk score values		
CHESS score (points)	5.65 (±1.37)	3.78 (±2.54)
SDASH score (points)	3.10 (±0.85)	2.13 (±1.41)
Patients’ characteristics		
Age (years)	53.87 (±13.18)	53.41 (±13.90)
Sex (female)	146 (66.1%)	257 (66.8%)
Aneurysm location (posterior circulation)	57 (25.8%)	98 (25.5%)
Hunt & Hess scale (grade 4–5)	125 (56.6%)	133 (34.5%)
BNI scale (grade 3–5)	138 (62.4%)	196 (50.9%)
Presence of IVH	141 (63.8%)	151 (39.2%)
Acute hydrocephalus	211 (95.5%)	246 (63.9%)

Abbreviations: *SD* standard deviation, *SAH* subarachnoid hemorrhage, *IVH* intraventricular hemorrhage, *A* maximum width between the two frontal horns, *a* internal width of the vault at level of A, *B* minimum width of the ventricles between caudate nuclei, *b* internal width of the vault at level of B, *C* greatest width of the third ventricle, *c* internal width of the vault at level of C, *D* maximum internal width of the vault. *CHESS* chronic hydrocephalus ensuing from SAH (subarachnoid hemorrhage) score, *SDASH* shunt dependency in SAH (subarachnoid hemorrhage)

Two hundred twenty-one individuals (36.5%) in the study cohort received permanent CSF diversion. Of them, five individuals (2.3%) underwent shunt revision surgery (placement of a shunt assistant device) due to shunt over-drainage during the mean post-SAH follow-up time of 41.7 months. Further, there were no cases requiring shunt removal due to over-drainage and missing/resolved chronic hydrocephalus.

**Fig. 3:**
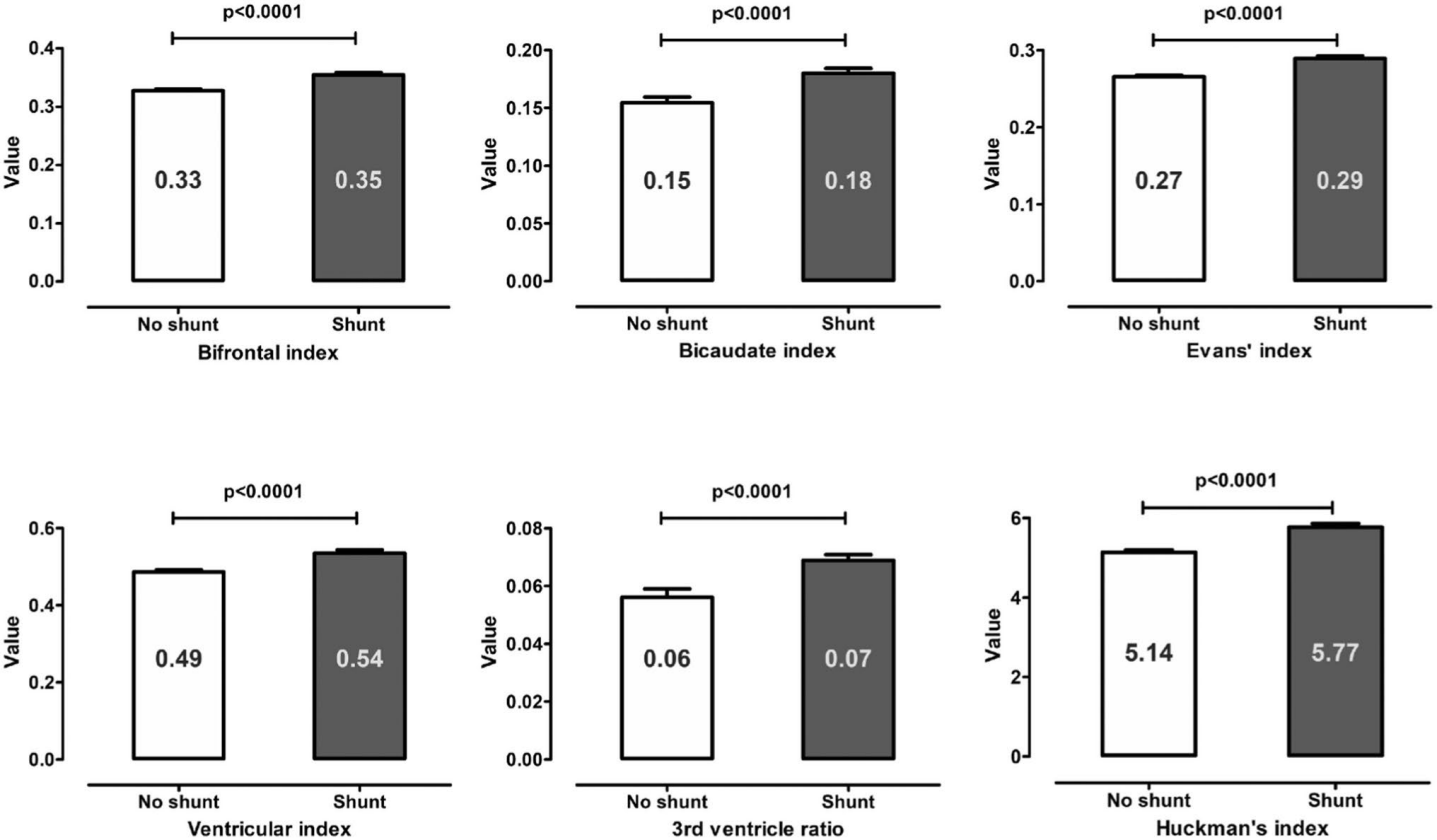
Univariate analysis of all ventricular indices and ratios’ comparing the values for patients with and without shunt placement. The values of all ventricular measurements are significantly higher in SAH patients undergoing shunt placement

In the univariate analysis, we found the values of all ventricular indices significantly higher in the group with shunt (all: *p*<0.0001) (see Fig. 3). In the subsequent ROC analysis, the values for the AUC for the ventricular parameters ranged from 0.622 to 0.662 (*p*<0.0001 for all indices) (see supplementary Table S2 in Online Supplements). In comparison, the AUC values for the shunt dependency risk scores were higher, with the CHESS score reaching the best diagnostic performance (AUC=0.713, 95% confidence interval [CI] 0.673–0.754, *p*<0.0001) followed by the SDASH score (AUC=0.693, 95% CI 0.651–0.734, *p*<0.0001).

Based on the above-mentioned ROC curves, a clinically relevant cut-off for the prediction of shunt placement was determined for all selected ventricular indices. Thereafter, a multivariate binary logistic backward regression analysis was applied. As the baseline predictor model, the CHESS score was included. In the final stepwise regression, we found only the Huckman’s index (cut-off at ≥ 6 cm, aOR=2.76, 95% CI 1.65–4.62, *p*<0.0001) to be associated with chronic hydrocephalus independent of the CHESS score (aOR=1.57 per point increase, 95% CI 1.39–1.77, *p*<0.0001) (see Table 2). Accordingly, both parameters were included in the new risk score for prediction of shunt dependency after SAH containing all original components of the CHESS score (acute hydrocephalus [4 points], Hunt and Hess=4–5 [1 point], IVH [1 point], aneurysm in the posterior circulation [1 point], and early cerebral infarction [1 point]) and the Huckman’s index (≥ 6 cm [2 points]).

**Table 2:** Multivariate binary logistic backward regression model

**Ventricular ratio/index**	**aOR (95% CI)**	***p*** **value**
*Step 1*		
CHESS score (per point increase)	1.57 (1.39–1.77)	**<0.0001**
Bifrontal ratio≥0.33	0.56 (0.30–1.04)	0.065
Bicaudate ratio≥0.16	1.09 (0.50–2.36)	0.828
Ventricular ratio≥0.52	0.76 (0.42–1.38)	0.370
3rd ventricle ratio≥0.05	1.35 (0.81–2.25)	0.256
Evans’ index≥0.25	1.71 (0.94–3.12)	0.081
Huckman’s index≥6.0 cm	2.69 (1.42–5.08)	**0.002**
*Step 2*		
CHESS score (per point increase)	1.57 (1.39––1.77)	**<0.0001**
Bifrontal ratio≥0.33	0.56 (0.30–1.04)	0.066
Ventricular ratio≥0.52	0.79 (0.48–1.29)	0.345
3rd ventricle ratio≥0.05	1.35 (0.81–2.26)	0.247
Evans’ index≥0.25	1.73 (0.95–3.13)	0.072
Huckman’s index≥6.0 cm	2.78 (1.57–4.91)	**<0.0001**
*Step 3*		
CHESS score (per point increase)	1.56 (1.38–1.76)	**<0.0001**
Bifrontal ratio≥0.33	0.57 (0.31–1.06)	0.074
3rd ventricle ratio≥0.05	1.23 (0.77–1.97)	0.388
Evans’ index≥0.25	1.73 (0.96–3.14)	0.070
Huckman’s index≥6.0 cm	2.56 (1.49–4.40)	**<0.0001**
*Step 4*		
CHESS score (per point increase)	1.57 (1.39–1.77)	**<0.0001**
Bifrontal ratio≥0.33	0.59 (0.32–1.08)	0.087
Evans’ index≥0.25	1.78 (0.99–3.21)	0.056
Huckman’s index≥6.0 cm	2.76 (1.65–4.62)	**<0.0001**

Abbreviations: *CHESS* chronic hydrocephalus ensuing from SAH (subarachnoid hemorrhage) score, *AUC* area under the curve, *ROC* receiver operating characteristics, *aOR* adjusted odds ratio, *CI* confidence interval. Significant values are in bold

The CHESS score was used as the baseline predictor. For all ventricular ratios/indices, the cut-off values, as determined by the AUC according to the ROC analysis, were used. Only Huckman’s index was independently associated with shunt dependency after stepwise regression

**Fig. 4:**
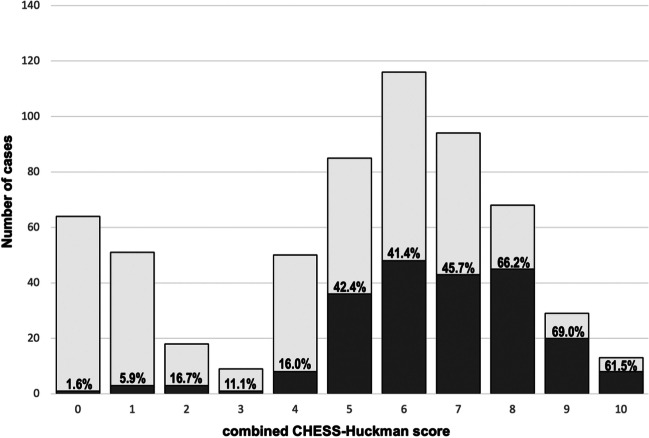
Percentage of SAH patients with shunt placement for every point on the combined CHESS-Huckman’s score. Overall, the higher the score, the more likely that the patient needs a permanent CSF diversion. Dark gray, shunt placement; light gray, no shunt placement

**Fig. 5:**
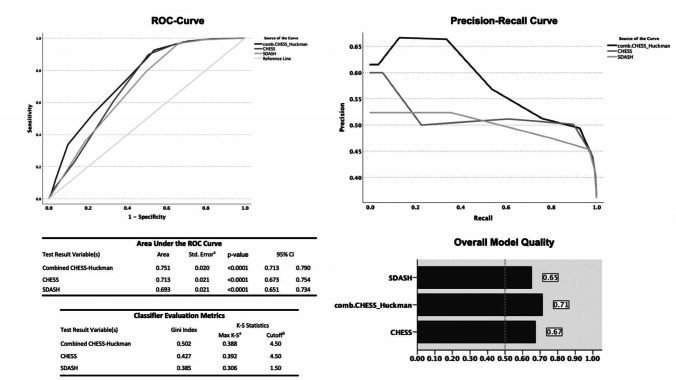
Diagnostic accuracy of the combined CHESS-Huckman’s score compared to the CHESS and SDASH scores in different models. The combined CHESS-Huckman’s score provides the best overall model quality with the largest AUC in the ROC and precision–recall analysis as well as the highest Gini index

This novel combined CHESS-Huckman score (0–10 points) (see Table 3) was then calculated for the whole cohort and thereafter correlated with the study endpoint. The more points the SAH individuals scored on the CHESS-Huckman scale, the higher the probability of shunt placement in the cohort, ranging from 1.6 to 69% (*p*<0.0001) (see Fig. 4). The diagnostic accuracy of the combined CHESS-Huckman score was also confirmed in the ROC analysis. The AUC for this novel risk score was 0.751 (95% CI 0.713–0.790, *p*<0.0001) higher than the AUC for the CHESS and SDASH scores (Fig. 5). The CHESS-Huckman score also performed better than other risk scores in the precision–recall curve. Finally, the Gini index (0.502 vs. 0.427 and 0.385) and the overall model quality (0.71 vs. 0.67 and 0.65) showed the novel combined CHESS-Huckman score’s superiority when compared with the CHESS and SDASH scores, respectively.

**Table 3:** Components and weights of parameters in the combined CHESS-Huckman score

**Parameter**	**Score weight**
Acute hydrocephalus	4
Initial clinical condition (Hunt & Hess grade 4–5)	1
Intraventricular hemorrhage	1
Ruptured aneurysm in the posterior circulation	1
Early cerebral infarction (within 72 h after SAH)	1
Huckman’s index ≥6.0 cm	2

When further analyzing the diagnostic performance of the combined CHESS-Huckman score in the cohort, we did not identify significant difference in the score values between shunted SAH individuals who did, or did not, require shunt revision surgery due to over-drainage (median 7.0 vs 7.0, *p*=0.414). We also analyzed the impact of baseline characteristics and common SAH complications (ICP increase and cerebral vasospasm) on the probability of false-positive and false-negative prediction of shunt dependency using the novel risk score. So, in the sub-cohort of SAH individuals with low combined CHESS-Huckman score values (0–4 points, *n*=192), 16 individuals (8.3%) underwent shunt placement despite a low-risk profile according to the score. The parameters significantly associated with the false-negative prediction of shunt placement in this “low shunt risk” sub-cohort were the development of acute hydrocephalus (shunt placement in 16.3% vs. 5.6%, *p*=0.032), intracerebral hemorrhage (15.5% vs. 5.2%, *p*=0.024), and cerebral vasospasm on DSA (20% vs. 6.2%, *p*=0.023) (see supplementary Table S3 in Online Supplements). In turn, in the sub-cohort of SAH individuals with high risk for shunt placement according to the combined CHESS-Huckman score (8–10 points, *n*=110), 37 patients (33.6%) did not develop shunt dependency during the post-SAH course. Here, only the development of cerebral vasospasm during SAH was significantly associated with the probability of false-positive prediction of shunt dependency in this “high shunt risk” SAH sub-population (no shunt in 15.4% vs 39.3% of cases, *p*=0.032).

## Discussion

Measurement of the ventricles in the initial stage of SAH can give significant information on the dependency on permanent CSF diversion in the later course of disease. With the current data, we showed that especially the Huckman’s index combined with the CHESS score gives a solid and accurate prediction model for the need of shunt placement after SAH.

High variability in the size of ventricle system has been reported in healthy individuals [47], and the patients’ age seems to be the major factor contributing to the ventricular morphology [8, 38]. A change in ventricle size is a common condition in different cerebral pathologies. It can manifest both as ventricle narrowing related to brain swelling or mass effect [46] and as ventricular enlargement as a consequence of idiopathic, post-inflammatory, or post-hemorrhagic hydrocephalus [35]. In particular, increase of ventricle size due to hydrocephalus is a common complication after aneurysm rupture necessitating an external CSF diversion in up to 97% of SAH individuals [16].

Despite extensive efforts in both experimental and clinical studies, the exact mechanisms by which acute and chronic hydrocephalus after SAH are caused remain somewhat elusive. It is thought that an inflammatory reaction lies at the base of both types of hydrocephalus, caused by blood outside of the vessels and in the subarachnoid space [6]. This inflammation could eventually lead to fibrosis and cell death, impeding the normal CSF flow and disrupting the blood–brain barrier [4, 6, 49]. Several biological markers have been found in these pathways, as mentioned by Chen et al. [6]. Besides causing a mechanical obstruction, blood clots are thought to play an important role in CSF hypersecretion, contributing to long-term hydrocephalus [30].

Post-hemorrhagic hydrocephalus is a frequent complication of SAH occurring at different stages of the disease [16, 23, 54]. After the acute and subacute phases (up to 14 days), hydrocephalus might persist despite weaning attempts, necessitating a permanent CSF diversion in about a third of SAH individuals [13, 33, 50, 55]. There is a substantial body of clinical evidence on the link between the risk of acute and chronic hydrocephalus. An almost sixfold increase in odds of shunt dependency was found in SAH patients with acute hydrocephalus, as was shown in a recent meta-analysis by Wilson et al [53]. However, for a more precise estimation of the risk of shunt dependency after SAH, knowledge of other relevant risk factors is of eminent importance. Several clinical research attempts have been conducted in this regard so far.

Since SAH is often accompanied by IVH, quantification of this blood inside the ventricles and its subsequent correlation with the risk of post-SAH hydrocephalus has been a topic of study in the last decades. In 1982, Graeb et al. developed a score to measure the amount of blood inside the ventricles and concluded that delayed hydrocephalus was rather an effect of the SAH than of IVH [19]. This score and its modified variant continue to be used as a tool for chronic hydrocephalus in SAH, although not necessarily intended for this population [9, 36]. Other patient and SAH characteristics repeatedly reported as independent predictors of shunt dependency are poor initial clinical condition, nosocomial meningitis, older age, worse mental function status on admission, anterior and middle cerebral artery aneurysms, coil embolization, vasospasm, and cerebral infarction [12, 40, 45, 57].

The risk scores commonly used in clinical practice allow cumulative assessment of the probability of a specific outcome parameter based on the relevant predictors. The earlier published risk scores like the CHESS [27] and SDASH [14] scores were constructed for the prediction of shunt dependency in SAH and have been validated in other SAH cohorts [56]. Recently, Garcia-Armengol et al. compared the Graeb, Hijdra [21], CHESS, and SDASH scores to predict shunt dependency in aneurysmal SAH patients [18]. While no significant differences were found for the SDASH and CHESS scores as well as the Graeb and Modified Graeb scores, the Hijdra score showed a lower predictive value.

Despite the above-mentioned clinical research, early and proper prediction of shunt dependency after SAH remains a topic of concern in clinical practice. The duration of external CSF diversion is an acknowledged risk factor for developing CSF infections [25, 44]. Additionally, ventriculitis is a dreaded complication with high in-hospital mortality and high rate of neurological deficits in the surviving patients [34]. In order to bring back the number of days of EVD usage and thus minimize the risk of meningitis/ventriculitis and their sequelae, it is of utmost importance to predict which patients are most likely to survive without shunt dependency and which will be dependent on permanent CSF diversion. Thus, the timely removal or internalization of the shunt could be planned, hopefully leading to a reduction in infection rates and their consequences. Moreover, timely and robust prediction of shunt dependency can potentially decrease treatment expenses due to shorter hospital stays and lower hospital readmission rates from rehabilitation centers for secondary shunt placement. These circumstances explain the continuing efforts to improve the diagnostic performance of the prediction tools for shunt dependency after SAH.

As we demonstrated with our data in a large, representative cohort, ventricular measurements on the admission CT scans of SAH patients are a reliable tool for early prediction of the need for shunt placement. The Huckman’s index proved to be an independent predictor for chronic hydrocephalus after SAH. After combining the previously described and validated CHESS score with the Huckman’s index, we found the novel combined score to be superior in diagnostic accuracy than the CHESS and SDASH scores for predicting the necessity of permanent CSF diversion in SAH patients.

The limitations of our study are its retrospective and monocentric design. Due to specific selection criteria aiming to rule out confounding effects on the study endpoint, we had to exclude a large number of patients from our final analyses. We believe, however, that despite this fact, our findings are representative of SAH patients as it remains the largest cohort up to date to compare different risk scores designed for SAH to predict shunt placement. As to the newly introduced combined CHESS-Huckman score, external validation is lacking at this point. This aspect is of particular importance, as the prediction scores previously introduced in the literature have still limited clinical utility. It is most likely due to missing or insufficient proof of diagnostic accuracy of these scores in external SAH cohorts. In our internal validation with different prediction models, however, we found it to be a robust and sensitive predictor of shunt placement in SAH patients. Finally, to address the potential selection bias related to the indications for shunt placement, a prospective evaluation of shunt dependency after SAH in the context of a multi-centric randomized trial is essential.

## Conclusions

Prediction of the need for permanent CSF diversion due to chronic hydrocephalus after intracranial aneurysm rupture can be accurately done by the known ventricular measurements at admission. Of these, the Huckman’s index has the highest value for predicting post-SAH chronic hydrocephalus. The combined CHESS-Huckman score provides an outstanding diagnostic accurateness for identifying SAH patients in our cohort who require permanent shunt placement. External validation of this novel score is, of course, necessary. We believe, nevertheless, it could provide a helpful tool for adequate and early selection of SAH patients at risk for chronic hydrocephalus.

## Supplementary information

ESM 1
