# Supplementary material for: The value of ventricular measurements in the prediction of shunt dependency after aneurysmal subarachnoid hemorrhage
Source: Acta Neurochir (Wien). 2023 May 2;165(6):1545–55. doi: 10.1007/s00701-023-05595-6 (PMC10227125; doi:10.1007/s00701-023-05595-6)
Supplement: Supplementary file 1 — ESM 1 [file 701_2023_5595_MOESM1_ESM.docx]

**Supplementary material**

**Table S1.** Components of the CHESS and SDASH scores.

| CHESS-score | *Points* | SDASH-score | *Points* |
| --- | --- | --- | --- |
| Hunt and Hess score ≥4 | 1 | Hunt and Hess score ≥4 | 1 |
| Aneurysm in posterior circulation | 1 | Barrow Neurological Index ≥3 | 1 |
| Acute hydrocephalus | 4 | Acute Hydrocephalus | 2 |
| Intraventricular hemorrhage | 1 |  |  |
| Early cerebral infarction | 1 |  |  |

**Table S2.** ROC analysis for all ventricular measurements.

| **Ventricular measurements/indices** | **AUC (95 % CI)** | **p-value** | **Cut-off** |
| --- | --- | --- | --- |
| **A** | 0.655 (0.608-0.702) | **<0.0001** | - |
| **B** | 0.656 (0.608-0.704) | **<0.0001** | - |
| **C** | 0.659 (0.612-0.706) | **<0.0001** | - |
| **Bifrontal ratio: A/a** | 0.648 (0.601-0.694) | **<0.0001** | >0.33 |
| **Bicaudate ratio: B/b** | 0.654 (0.606-0.702) | **<0.0001** | >0.16 |
| **Ventricular ratio: B/A** | 0.622 (0.572-0.671) | **<0.0001** | >0.52 |
| **Third ventricle ratio: C/c** | 0.661 (0.614-0.708) | **<0.0001** | >0.05 |
| **Evans’ index: A/D** | 0.662 (0.616-0.709) | **<0.0001** | >0.25 |
| **Huckman’s index: A+B** | 0.662 (0.615-0.709) | **<0.0001** | >6.0 |

Based on the AUC values a clinically relevant cut-off was determined for the ventricular ratios and indices.

Abbreviations: ROC= receiver operating characteristics, AUC= area under the curve, aOR= adjusted odds ratio, CI= confidence interval, **A=** maximum width between the two frontal horns, a= internal width of the vault at level of A, B= minimum width of the ventricles between caudate nuclei, b= internal width of the vault at level of B, C= greatest width of the third ventricle, c= internal width of the vault at level of C, D= maximum internal width of the vault. Significant values are in **bold.**

**Table S3.** Univariate analysis of the risk factors for shunt placement in the sub-cohorts with **low** (<5 points) and **high** (>7 points) risk profiles according to the novel combined CHESS-Huckman score.

| **Parameter** | **Low Risk (combined CHESS-Huckman score 0 – 4 points)** | | | **High Risk (combined CHESS-Huckman score 8 – 10 points)** | | |
| --- | --- | --- | --- | --- | --- | --- |
|  | *FNR w/o RF* | *FNR w. RF* | *p-value* | *FPR w/o RF* | *FPR w. RF* | *p-value* |
| **Age (>55 years)** | 8.9% | 8.1% | 0.783 | 37.7% | 26.8% | 0.299 |
| **Sex (female)** | 4.4% | 10.5% | 0.179 | 29.3% | 36.2% | 0.534 |
| **Acute hydrocephalus** | 5.6% | 16.3% | **0.032** | 33.6% | 33.6% | 1.000 |
| **IVH** | 8.0% | 11.1% | 0.650 | 33.3% | 33.7% | 1.000 |
| **ICH** | 5.2% | 15.5% | **0.024** | 32.4% | 36.1% | 0.830 |
| **Hunt&Hess grade 4-5** | 7.2% | 16.0% | 0.137 | 37.5% | 32.6% | 0.635 |
| **Treatment modality (clipping)** | 5.9% | 11.1% | 0.204 | 35.5% | 29.4% | 0.663 |
| **Aneurysm location (PC)** | 8.5% | 7.1% | 1.000 | 27.1% | 41.2% | 0.157 |
| **ICP increase** | 7.3% | 9.6% | 0.561 | 34.4% | 33.3% | 1.000 |
| **Cerebral vasospasm on DSA** | 6.2% | 20.0% | **0.023** | 39.3% | 15.4% | **0.032** |

Abbreviations: FNR= false negative rate (percentage of individuals with shunt despite low risk profile on the score); w/o= without; w.= with; RF= risk factor; FPR= false positive rate (percentage of individuals without shunt despite high risk profile on the score); IVH= intraventricular hemorrhage; ICH= intracerebral hemorrhage; PC= posterior circulation; ICP= intracranial pressure; DSA= digital subtraction angiography.
